# Bilateral Ovarian Steroid Cell Tumor With One-Year Disease-Free Follow-Up: A Case Report

**DOI:** 10.7759/cureus.99466

**Published:** 2025-12-17

**Authors:** Luis Muñoz, Karla A Fabiani, Carlos A Cardenas, Luis A Barrera, Guido Panchana

**Affiliations:** 1 Department of Gastrointestinal Surgery, Centro Médico Muñoz y Andrade, Quito, ECU; 2 Department of Surgery, Universidad de Especialidades Espiritu Santo, Guayaquil, ECU; 3 Department of Surgery, Medical Center Dykai, Guayaquil, ECU; 4 Department of Surgical Oncology, Instituto Oncológico Nacional Dr. Juan Tanca Marengo, Sociedad de Lucha Contra el Cancer (SOLCA), Guayaquil, ECU; 5 Department of Surgery, Instituto Oncológico Nacional Dr. Juan Tanca Marengo, Sociedad de Lucha Contra el Cancer (SOLCA), Guayaquil, ECU

**Keywords:** adjuvant chemotherapy, bilateral ovarian tumor, fertility preservation, immunohistochemistry, ovarian steroid cell tumor, sex cord–stromal tumor

## Abstract

Ovarian steroid cell tumors are rare sex cord-stromal neoplasms that may produce androgens and present with nonspecific gynecologic symptoms. Bilateral involvement is particularly uncommon and raises important diagnostic and therapeutic challenges in young women. We describe the case of a 25-year-old woman with bilateral adnexal masses who was evaluated with cross-sectional imaging and surgery, leading to the diagnosis of an ovarian steroid cell tumor. Definitive management was planned in a multidisciplinary setting and included surgical resection, adjuvant systemic treatment, and structured clinical, biochemical, and radiologic surveillance. The patient has remained disease-free on follow-up. This case underscores the importance of considering steroid cell tumor in the differential diagnosis of solid ovarian masses in reproductive-age patients, the central role of histopathology and immunohistochemistry in confirming the diagnosis, and the need to balance oncologic safety with fertility and endocrine considerations in young women with rare ovarian tumors.

## Introduction

Steroid cell tumors (SCTs) of the ovary are rare sex-cord stromal neoplasms, accounting for less than 0.1% of all ovarian tumors [1]. They are most commonly unilateral and typically diagnosed in reproductive-age or perimenopausal women. Clinical manifestations depend on hormonal activity, with hyperandrogenism, menstrual irregularities, virilization, or, less frequently, estrogenic or cortisol-related symptoms [2].

Bilateral involvement is exceptionally uncommon, reported in less than 6% of cases, and is usually associated with more aggressive biological behavior or underlying genetic predisposition [3]. Due to their rarity, optimal diagnostic strategies, prognostic factors, and follow-up recommendations remain poorly defined. Most available evidence comes from isolated case reports or small series, limiting the ability to standardize management.

We present a case of bilateral ovarian SCT in a 25-year-old woman, successfully managed with surgical resection, with one year of disease-free follow-up. This report highlights the diagnostic challenges, intraoperative considerations, and postoperative surveillance required for these rare tumors. Our case contributes valuable clinical information to the limited global literature on bilateral SCTs and reinforces the importance of early recognition and tailored management.

## Case presentation

A 25-year-old woman, G2P2, with no significant medical or oncologic history, presented with intermittent lower abdominal pain and progressive abdominal distension over several months. She denied virilization symptoms, menstrual irregularities, or constitutional changes. Physical examination revealed a palpable abdominopelvic mass without signs of acute abdomen.

**Figure 1:**
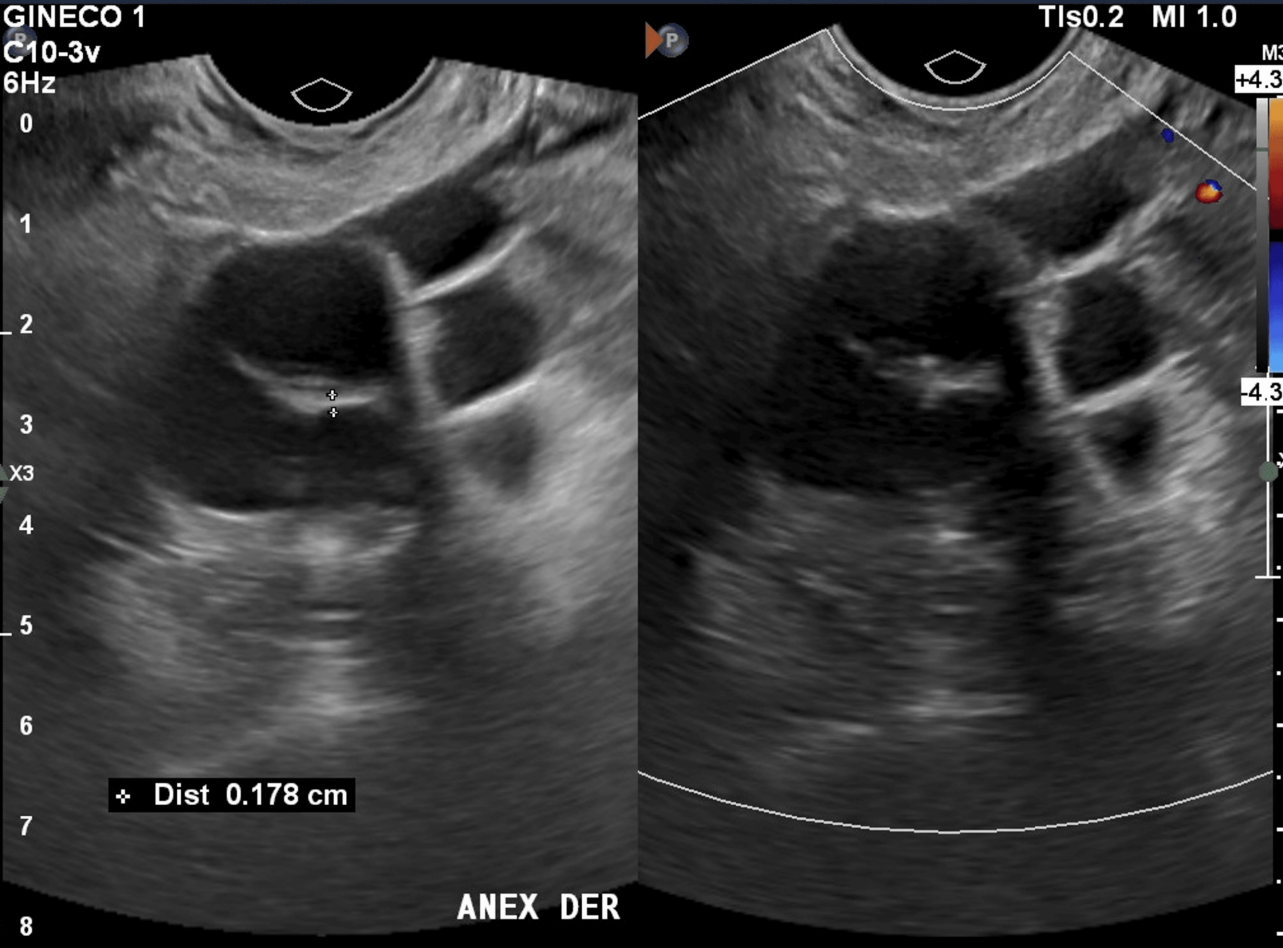
Transvaginal pelvic ultrasound showing a right ovarian mass with well-defined borders and heterogeneous echotexture.

**Figure 2:**
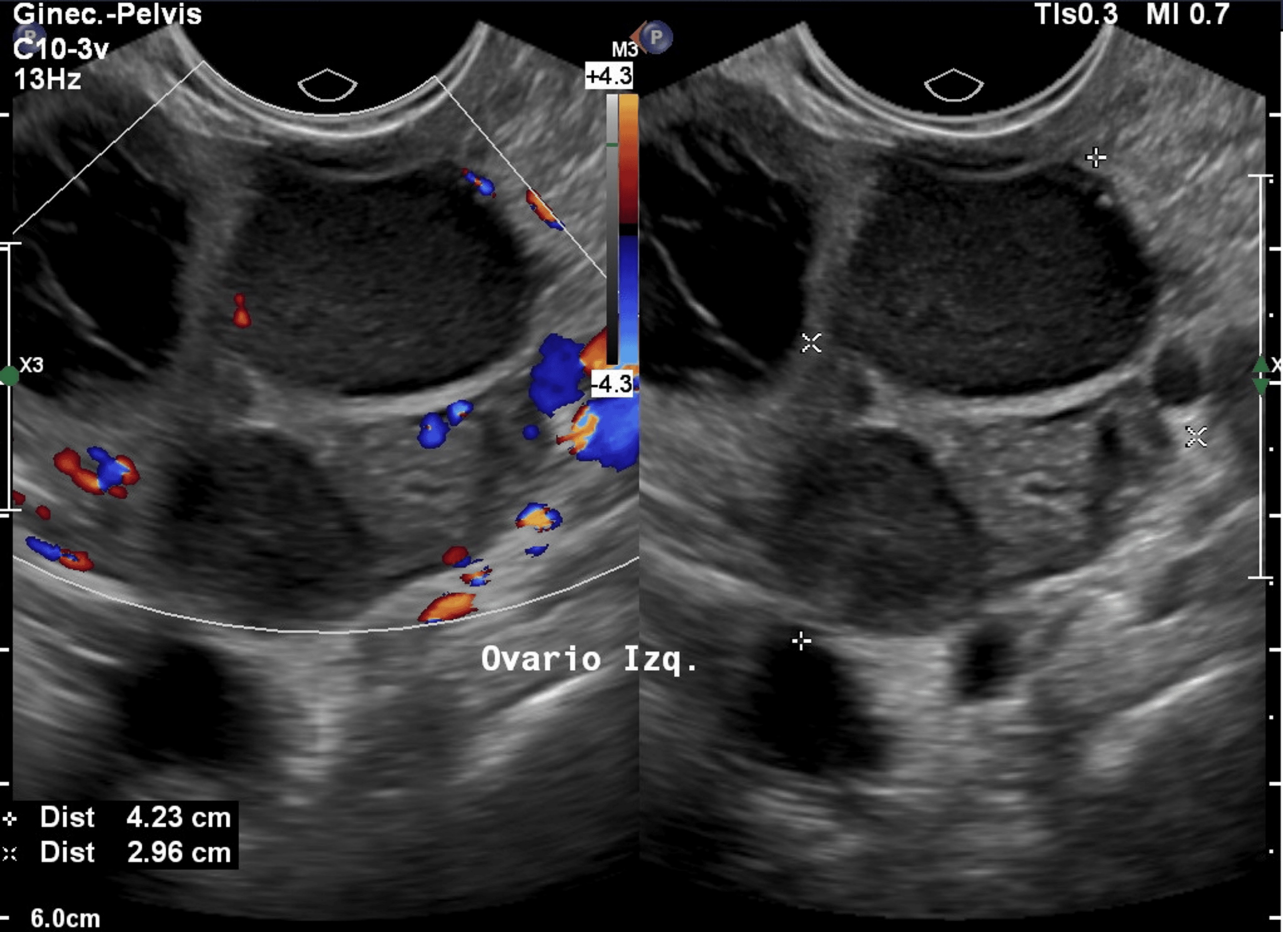
Transvaginal pelvic ultrasound demonstrating a predominantly solid left ovarian mass with heterogeneous echotexture and clearly defined margins.

Pelvic ultrasound demonstrated bilateral ovarian solid masses with well-defined borders and heterogeneous echotexture (Figures 1, 2).

**Figure 3:**
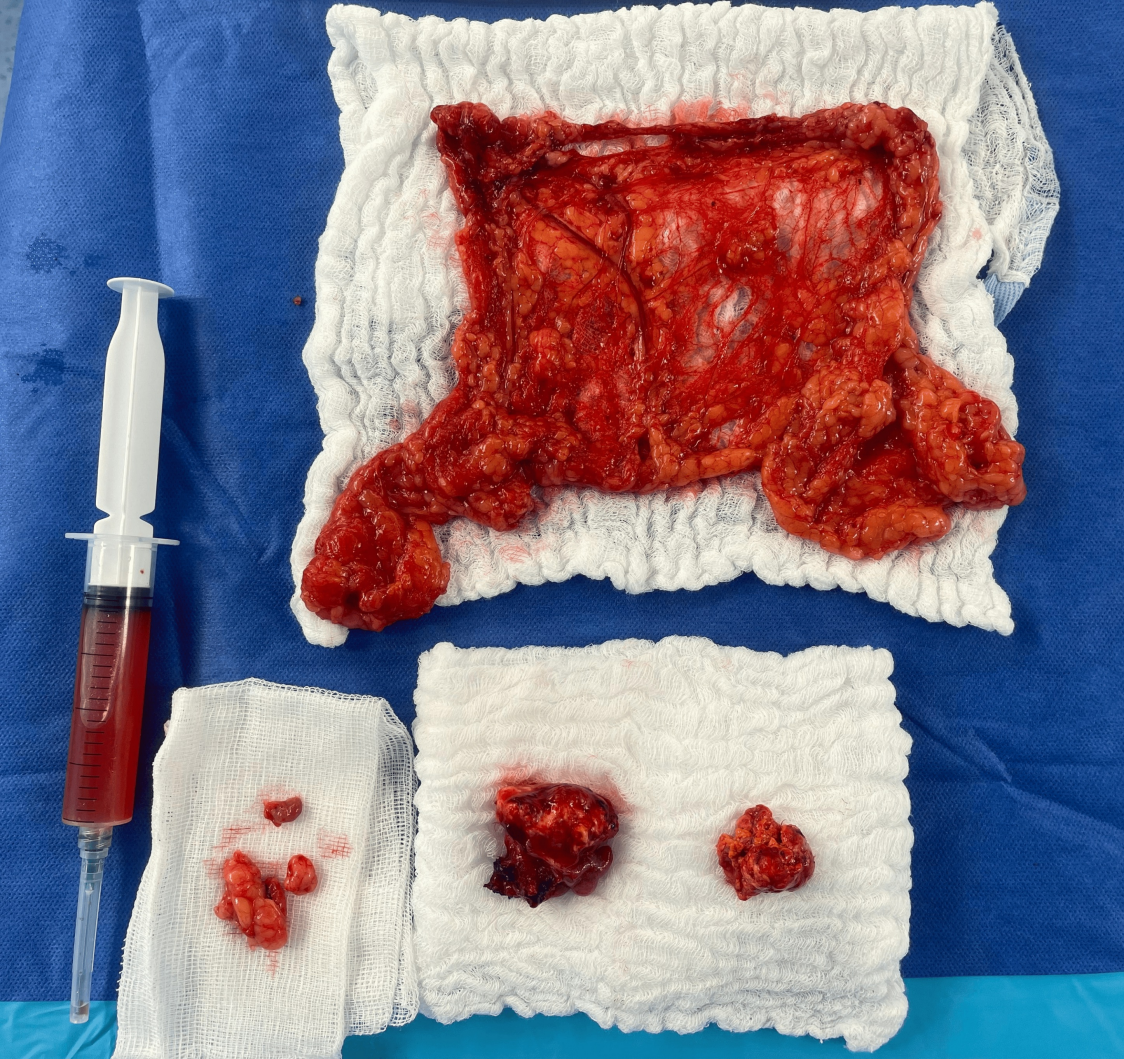
Gross surgical specimens of omentum and bilateral ovaries Omental specimen measuring 45 × 17.5 × 0.8 cm, yellow-brown and soft, without nodules or implants. The right ovary was almost entirely replaced by a steroid cell tumor measuring 4.2 × 2.7 × 1.6 cm, with capsular rupture. The left ovary showed a steroid cell tumor measuring 3.5 × 3.2 × 1.8 cm, also with capsular rupture. Regional lymph nodes (0/7) and peritoneal washing were negative for malignancy.

Serum tumor markers, including CA-125, CA-19.9, alpha-fetoprotein (AFP), beta-human chorionic gonadotropin (β-hCG), and lactate dehydrogenase (LDH), were within normal limits. Hormonal profile revealed mildly elevated total testosterone (82 ng/dL; reference <60 ng/dL) with normal dehydroepiandrosterone sulfate (DHEA-S) and cortisol levels. The patient underwent exploratory laparotomy with bilateral salpingo-oophorectomy. Intraoperatively, both ovaries were enlarged, well-circumscribed, and without capsular rupture. No ascites, peritoneal implants, or lymphadenopathy were observed. Histopathological examination revealed a SCT measuring 4.2 × 2.7 × 1.6 cm in the right ovary and 3.5 × 3.2 × 1.8 cm in the left ovary, with capsular rupture of unknown timing, consistent with pT1c2 disease. Gross surgical specimens are shown in Figure 3.

**Figure 4:**
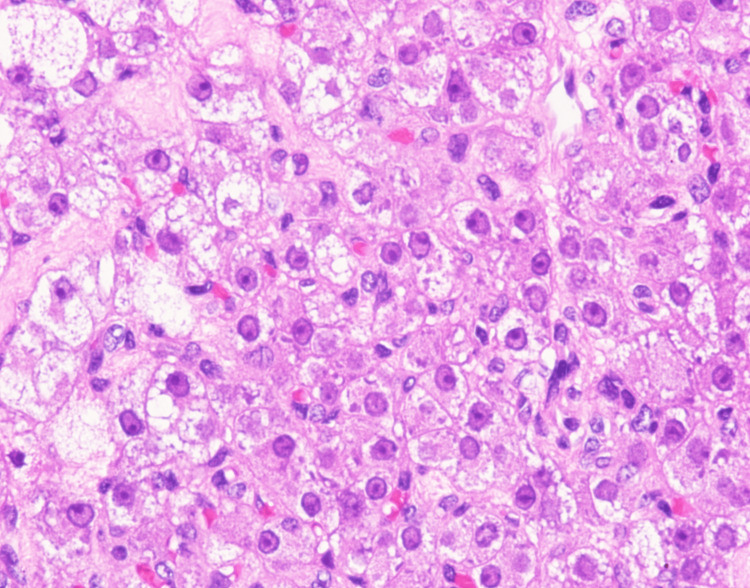
Histologic section showing polygonal cells with abundant eosinophilic cytoplasm Histological section demonstrating sheets of polygonal tumor cells with abundant eosinophilic cytoplasm and centrally located nuclei, consistent with ovarian steroid cell tumor. Hematoxylin and eosin (H&E), 40×.

**Figure 5:**
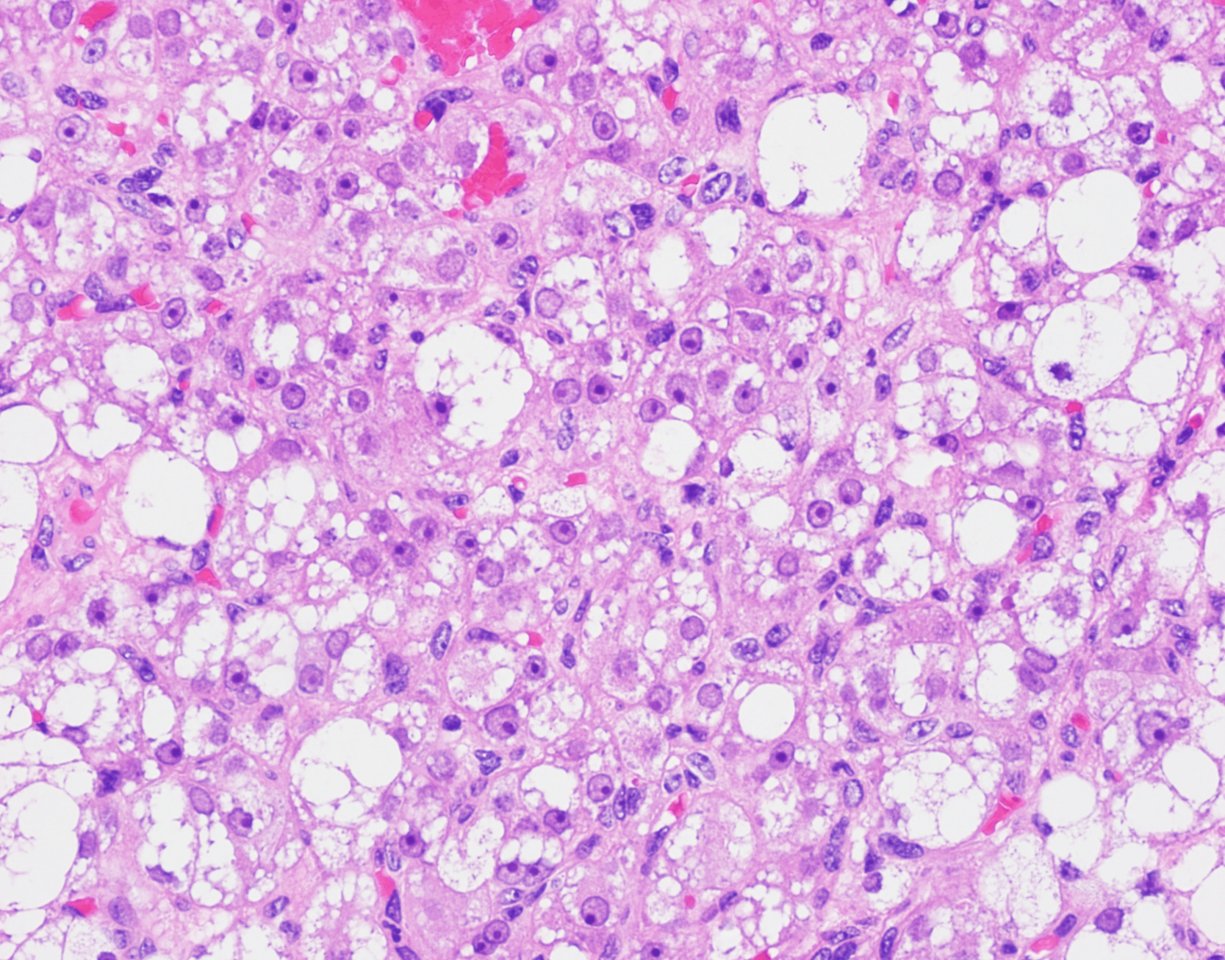
Histologic section showing clear to vacuolated tumor cells Microscopic section showing diffuse growth of clear to vacuolated tumor cells with prominent nucleoli. These features support the diagnosis of ovarian steroid cell tumor. Hematoxylin and eosin (H&E), 20×.

**Figure 6:**
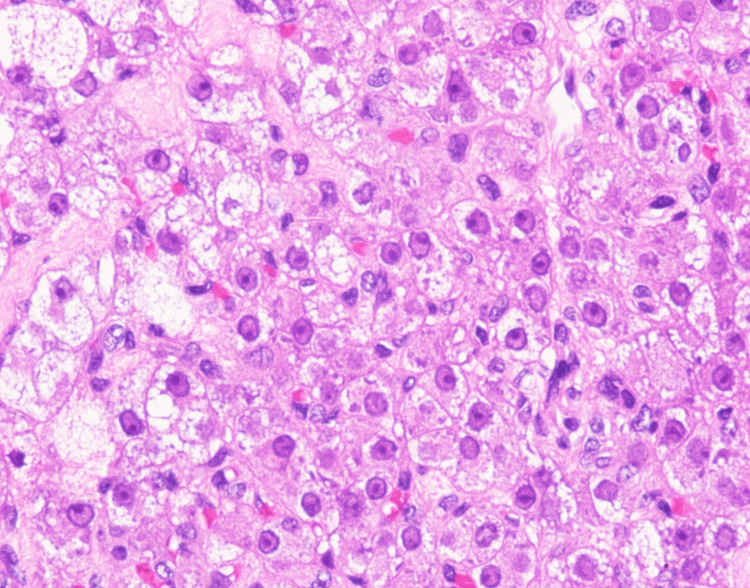
Heterogeneous morphology of lipid-rich and lipid-poor tumor cells Histological section highlighting the heterogeneous appearance of lipid-rich and lipid-poor tumor cells, demonstrating the characteristic variability seen in steroid cell tumors. Hematoxylin and eosin (H&E), 10×.

Microscopy demonstrated sheets of polygonal cells with eosinophilic to vacuolated cytoplasm, minimal atypia, and absence of Reinke crystals. Representative microscopy is shown in Figures 4-6.

**Figure 7:**
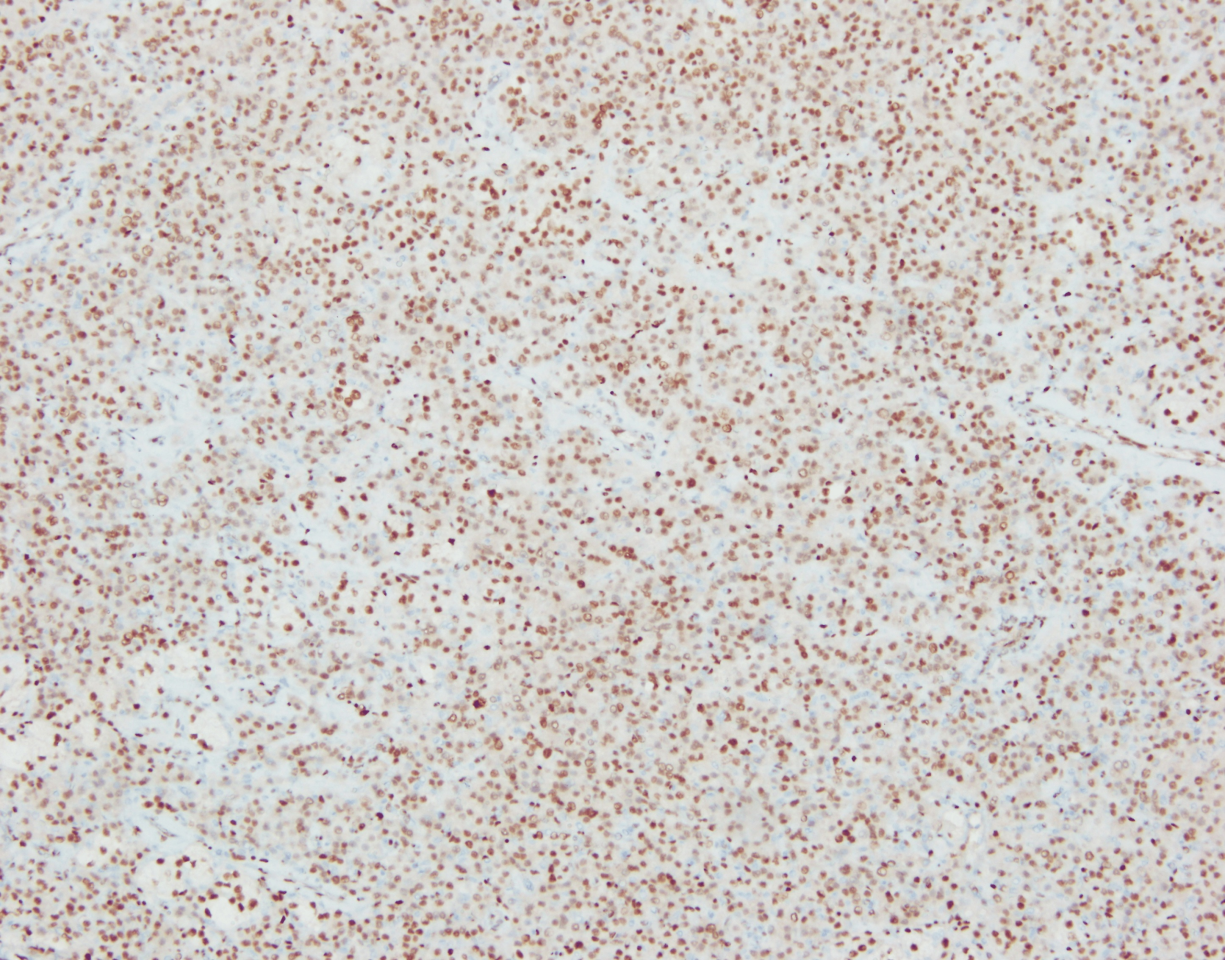
Immunohistochemical staining showing diffuse nuclear positivity for androgen receptor This supports the steroidogenic profile of the tumor.

**Figure 8:**
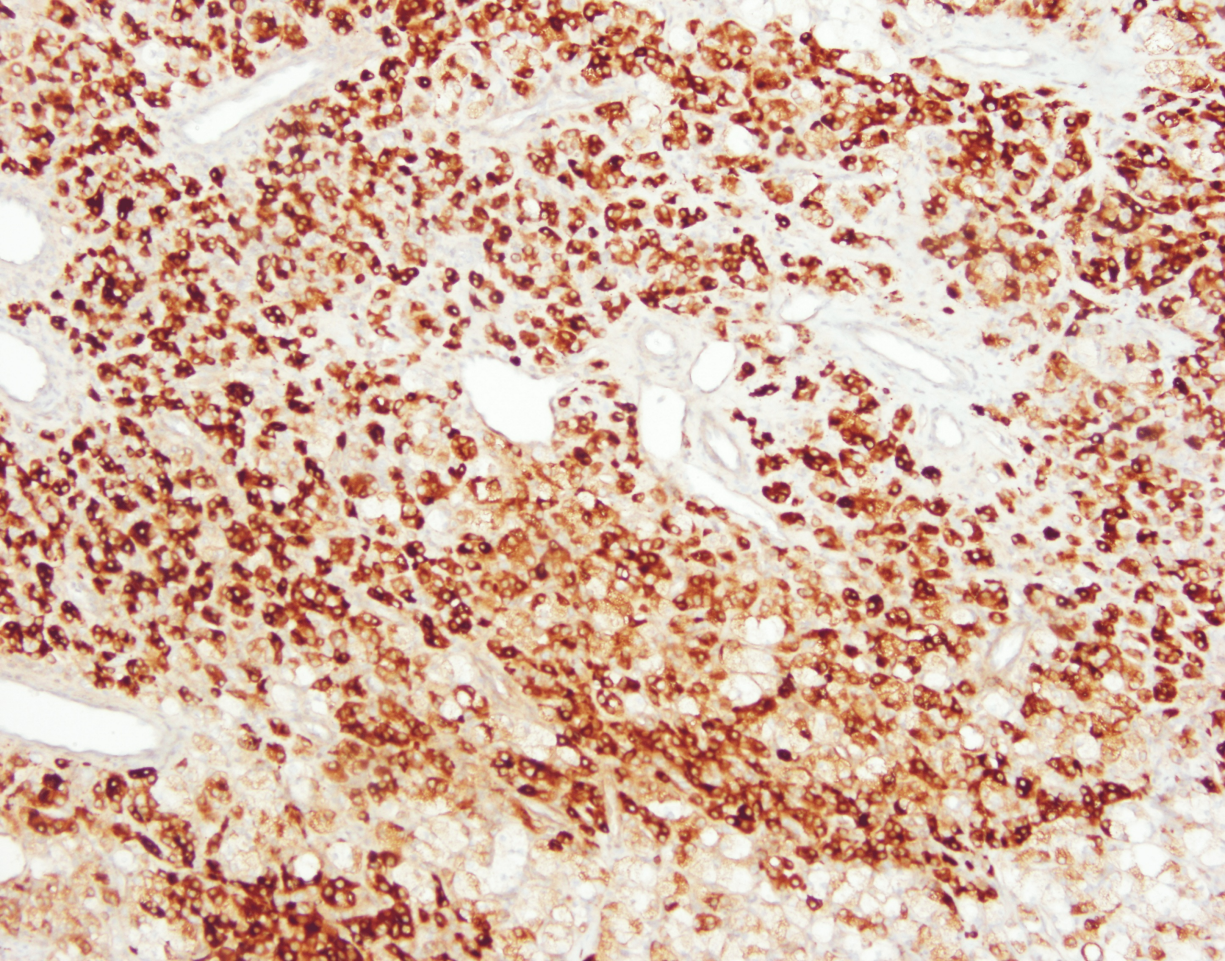
Immunohistochemical staining showing strong and diffuse cytoplasmic positivity for inhibin, a sensitive marker for sex cord-stromal tumors

**Figure 9:**
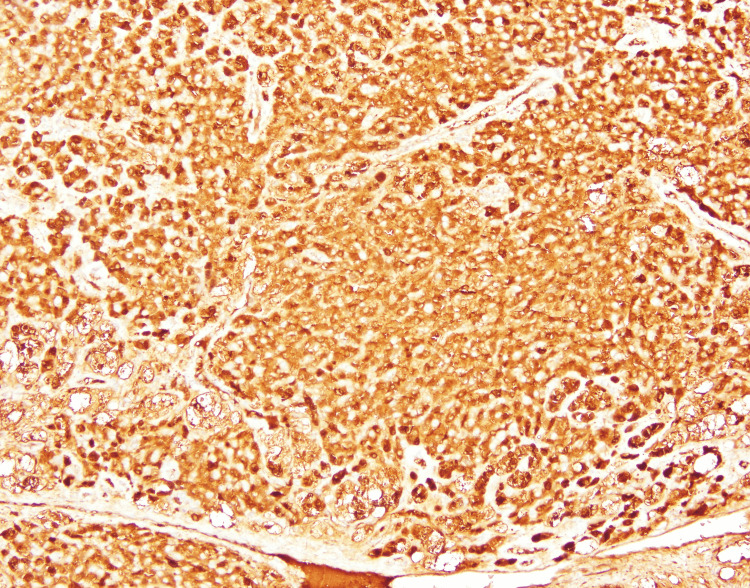
Immunohistochemical staining showing diffuse nuclear and cytoplasmic positivity for calretinin This further supports the diagnosis of steroid cell tumor.

Immunohistochemistry demonstrated inhibin-α (+), calretinin (+), Melan-A (+), and cytokeratin (−), confirming the diagnosis of bilateral ovarian SCT, not otherwise specified. Additional markers are shown in Figures 7-9.

**Figure 10:**
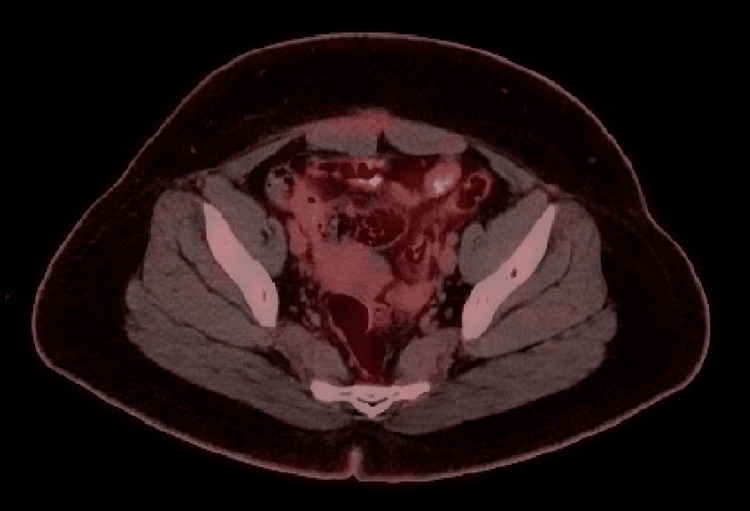
Follow-up PET-CT scan showing no evidence of metabolic disease Follow-up PET-CT scan performed after treatment, demonstrating no pathological radiotracer uptake in the abdominopelvic cavity, consistent with absence of active disease or recurrence.

Postoperative recovery was uneventful. Serum testosterone normalized within four weeks. At the one-year follow-up, the patient remained asymptomatic, with a stable hormonal profile and no radiologic evidence of recurrence. Follow-up imaging is shown in Figure 10.

## Discussion

SCTs of the ovary are rare sex cord-stromal neoplasms, accounting for less than 0.1% of all ovarian tumors. They are traditionally classified into three subtypes: stromal luteoma, Leydig cell tumor, and SCT not otherwise specified (NOS), which represents approximately 60% of cases [1]. SCT-NOS typically affects women in the third to fifth decades of life and is usually unilateral. Bilateral SCT-NOS, as observed in our patient, is exceptionally uncommon, occurring in fewer than 6% of reported cases [2].

Clinically, SCTs are characterized by hormone production, most commonly androgens, leading to virilization in approximately 50% of patients. However, a significant proportion remains asymptomatic or presents with non-specific abdominal symptoms, which may delay diagnosis [3]. In our case, the patient exhibited only mildly elevated testosterone without overt virilization, consistent with the lower functional activity described in a subset of SCT-NOS tumors.

Radiologic findings can suggest the diagnosis but are not pathognomonic. SCTs typically appear as solid, well-circumscribed, T2-hypointense masses on MRI with variable contrast enhancement, reflecting their lipid-rich content [4]. These features were present bilaterally in our patient, raising suspicion for a steroid-producing neoplasm. Tumor markers, including CA-125 and AFP, are usually normal and therefore not clinically helpful, as seen in this case.

Histopathological evaluation is essential for definitive diagnosis. Classic features include polygonal cells with eosinophilic or vacuolated cytoplasm, low mitotic activity, and absence of Reinke crystals, findings that closely matched our patient’s tumor morphology [5]. Immunohistochemistry is crucial for confirmation, with inhibin, calretinin, and Melan-A being the most consistently positive markers in SCT-NOS [6]. The combination of inhibin and calretinin positivity in our case strongly supported the diagnosis.

Although most SCT-NOS tumors behave benignly, approximately 20% may display malignant potential. Histologic predictors of aggressive behavior include size greater than 7 cm, necrosis, hemorrhage, high mitotic rate (>2 mitoses/10 high-power field (HPF)), and significant nuclear atypia [7]. Notably, despite the bilateral involvement and size of the masses, our patient’s tumors exhibited none of the worrisome histologic features, suggesting a favorable prognosis.

Surgical management remains the cornerstone of treatment. For women who have completed childbearing, bilateral salpingo-oophorectomy is recommended, with staging procedures considered based on intraoperative findings. Given the absence of capsular rupture, ascites, or metastatic spread in our case, no additional staging procedures were required. The role of lymphadenectomy is generally limited, as nodal involvement is exceedingly rare [8].

Follow-up care focuses on clinical evaluation, hormonal monitoring, and imaging as indicated. Normalization of serum testosterone levels within weeks after surgery is a strong indicator of complete tumor resection and a favorable biochemical response [9]. Our patient demonstrated full hormonal normalization and no evidence of recurrence at one-year follow-up, which aligns with the expected clinical course for SCT-NOS lacking malignant features.

This case is notable for its bilateral presentation, mild hormonal elevation, and benign histologic profile-all uncommon findings in combination. The inclusion of detailed histopathology and imaging highlights the diagnostic complexity and underlines the importance of a multidisciplinary approach.

## Conclusions

SCTs of the ovary are rare neoplasms with highly variable hormonal activity and a broad clinical spectrum. Bilateral involvement is exceptional and may pose diagnostic uncertainty. In this case, imaging, hormonal assessment, and detailed histopathology, including a characteristic immunohistochemical profile, were essential for establishing the diagnosis.

Complete surgical excision remains the cornerstone of treatment and is usually curative in tumors lacking high-risk pathological features. The patient experienced full biochemical normalization and remains disease-free at one year of follow-up, underscoring the favorable prognosis of benign SCTs. Continued surveillance is recommended to ensure early detection of the rare instances of recurrence.
